# VMSbase: An R-Package for VMS and Logbook Data Management and Analysis in Fisheries Ecology

**DOI:** 10.1371/journal.pone.0100195

**Published:** 2014-06-16

**Authors:** Tommaso Russo, Lorenzo D'Andrea, Antonio Parisi, Stefano Cataudella

**Affiliations:** 1 Laboratory of Experimental Ecology and Aquaculture – Department of Biology – “Tor Vergata” University of Rome, via della Ricerca Scientifica s.n.c., Rome, Italy; 2 Department of Economics and Finance - Faculty of Economics – “Tor Vergata” University of Rome, Rome, Italy; Institut Maurice-Lamontagne, Canada

## Abstract

VMSbase is an R package devised to manage, process and visualize information about fishing vessels activity (provided by the vessel monitoring system - VMS) and catches/landings (as reported in the logbooks). VMSbase is primarily conceived to be user-friendly; to this end, a suite of state-of-the-art analyses is accessible via a graphical interface. In addition, the package uses a database platform allowing large datasets to be stored, managed and processed vey efficiently. Methodologies include data cleaning, that is removal of redundant or evidently erroneous records, and data enhancing, that is interpolation and merging with external data sources. In particular, VMSbase is able to estimate sea bottom depth for single VMS pings using an on-line connection to the National Oceanic and Atmospheric Administration (NOAA) database. It also allows VMS pings to be assigned to whatever geographic partitioning has been selected by users. Standard analyses comprise: 1) métier identification (using a modified CLARA clustering approach on Logbook data or Artificial Neural Networks on VMS data); 2) linkage between VMS and Logbook records, with the former organized into fishing trips; 3) discrimination between steaming and fishing points; 4) computation of spatial effort with respect to user-selected grids; 5) calculation of standard fishing effort indicators within Data Collection Framework; 6) a variety of mapping tools, including an interface for Google viewer; 7) estimation of trawled area. Here we report a sample workflow for the accessory sample datasets (available with the package) in order to explore the potentialities of VMSbase. In addition, the results of some performance tests on two large datasets (1×10^5^ and 1×10^6^ VMS signals, respectively) are reported to inform about the time required for the analyses. The results, although merely illustrative, indicate that VMSbase can represent a step forward in extracting and enhancing information from VMS/logbook data for fisheries studies.

## Introduction

The exploitation of living resources by fisheries is a complex game played by different actors, each having distinct dynamics and patterns in space and time. Resources and environment are traditional objects of investigation and modelling, whereas an exhaustive analysis of fishing effort has been hampered by the lack of data about fishing fleet activities in space and time and of proper tools to analyse them. This situation has gradually changed since 2006, with the introduction of the Vessel Monitoring System (VMS), that is a collection system of fishing activity data in space and time. This represented a revolution in fisheries science, as it allowed the development of models and assessments in which fishing effort is explicitly taken into account. Each fishing vessel is equipped with a blue box that periodically sends signals (pings) containing data about position, speed and course of the vessel. Each signal sent by the blue box is a VMS ping.

In the past few years, the analysis of VMS and logbook data has received considerable attention even in terms of dedicated methodologies and applications [1]–[4]. The reason for this interest is the fact that VMS data allows fishing effort to be resolved as a function of its heterogeneity in space and time, and therefore allows fisheries impact on living resources and environment to be evaluated. In this context, different platforms for VMS data pre-processing and analysis have been proposed, the most structured version being by the VMStools package [5]. The present paper proposes what can be considered further progress in this field: a new integrated suite of tools with a step-by-step procedure for processing data starting from the raw VMS/logbook format and performing quantitative analyses of fishing effort. The name of this new suite is VMSbase, as one of its main and innovative skills is represented by the possibility of efficiently storing and managing large datasets.

**Figure 1:**
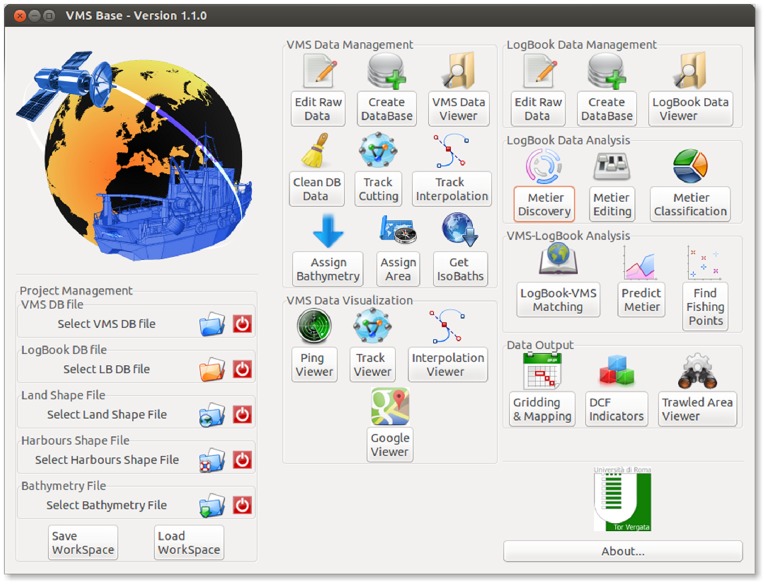
Main interface of the VMSbase package. It is organized into groups of icons that allow access to different functions that operate on different input data (VMS, logbooks, or a combination of the two). The “Project Management” panel on the bottom left side allows setting the files for the work session (and also saving and loading the workspace). The “VMS Data Management” and “Logbook Data Management” provide access to functions and routines for the analysis of, respectively, VMS and Logbook data. These two flows converge in the analyses provided in the “VMS-Logbook Analysis” panel. Finally, the main graphical and numerical outputs can be produced by the tools in the “Data Output” panel at the bottom-right side of the main interface.

The idea underlying VMSbase is based on the following factors (in order of importance): 1) fisheries data (such as VMS and logbook) can be characterized by different formats (e.g. ICES countries adopt a particular format) and convey peculiarities of the relative fishery in which they are collected, so that the ones surveyed in the Mediterranean can be distinguished from those of other areas (e.g. the North Sea), leading to the need for appropriate processing algorithms [6], [7]. VMSbase is therefore aimed to satisfy the need for appropriate methodologies to analyse fishing effort in the Mediterranean Sea, as recently advocated by [7]; 2) VMSbase implements a set of desirable (e.g. the linkage of VMS data to key environmental data such as bathymetry) or necessary tools (e.g. a database manager to efficiently manage typical VMS/Logbook datasets and a friendly windows interface, see Figure 1). Moreover, it employs some methods which are state-of-the-art (i.e. the interpolation algorithm [8] and the use of Artificial Neural Network to predict the métiers (homogeneous categories of fishing activities in terms of target species or assemblages, fishing area and fishing season, see [9] when logbook data are not available); 3) while retaining the possibility to use the command line interface, the graphical interface allows a very easy point-and-click approach to the analysis.

This paper is organized so as to illustrate the main features and potentiality of VMSbase. Sample datasets were thus used for a subset of the Italian fleet. Hence, the following sections are dedicated to the description of a typical processing path for these data. The analyses carried out for this study are not intended to provide indications or conclusions about the activity of the Italian fleet.

## Materials and Methods

### Description of the VMSbase Package

VMSbase was basically developed as part of the activity of the Italian National Program for Data Collection of the Fisheries Sector, during the years 2009–2012. It was decided to write VMSbase in R [10] as this is the most widely used software environment in this field, it is free, and offers a wide series of useful add-on libraries for dealing with spatial data. VMSbase can be downloaded from a dedicated website (www.vmsbase.org). This website contains a link to the public repository (hosted by GitHub - https://github.com/) from which the self-installing R add-on file and some additional resources (i.e. maps for mainland, harbours and some commonly used sea partitioning such as Geographic Sub Areas, ICES rectangles or Large Marine Ecosystems) can be downloaded, and also provides supporting contacts, news about the progressive update of the software, and the possibility of being involved in the development project. After installation, VMSbase should be run as a common R library. It is enough to load the package and launch the main interface of the package (Figure 1) using just two command lines.

The main interface is designed to show all the modules of the package, grouped by typology/level of the analysis or by type of data (VMS/Logbook) to be handled. A first point to be stressed is that each kind of elaboration can be carried out independently. In other words, in the same session, the user can decide to perform two or more different analyses (e.g. Assign Bathymetry and LogBook-VMS Matching on two different datasets) in whatever order, as long as the correct type of data has been already loaded. Each object managed by the functions of the package has an own independent existence of its own, and can be saved and loaded separately. However, this does not rule out the possibility of progressively and continuously processing a given dataset from the input to produce a defined output. In this way, one can load a given VMS or Logbook file at the beginning, via the “Project Management” panel on the left side of the main interface (Figure 1) and then perform different analyses without re-calling it, as the package will use that file as default for each analysis.

### Sample Datasets

**Figure 2:**
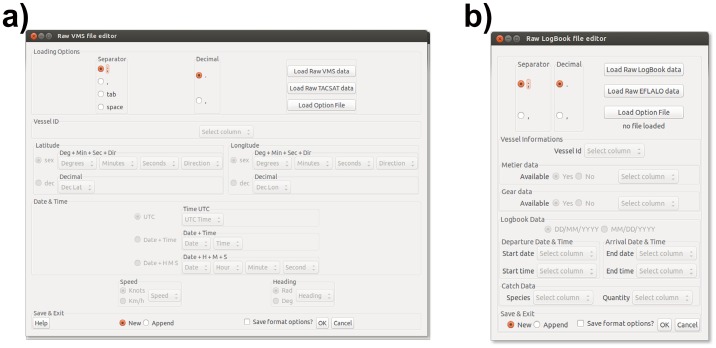
VMSbase interfaces for: a) VMS and b) Logbook data loading. While a series of data format options is available in both cases, these interfaces also allow the selected configurations to be saved.

VMSbase is equipped with two user-friendly interfaces that allow VMS and Logbook data to be loaded in a wide variety of formats (Figure 2). Obviously, data to be uploaded must contain some mandatory columns, although it is not necessary for the names of these columns to conform to a standard. The upload functions can actually manage data with different field separators (comma, semicolon and tabulation) and different formats for critical data (i.e. time and spatial coordinates). However, the graphical frame contains buttons to directly load in the package data formatted in the standard EFLALO/TACSAT2 formats for the purpose of compatibility purposes with VMStools and the North Sea scientific community (Figure 2b). Finally, the specific options used to load a specific dataset can be saved and used for similar datasets, making this step fast and secure. If the user is interested in running the loading function iteratively (i.e. when a large number of datasets have to be loaded), this can be done using command lines. The datasets used in the present paper were obtained from real datasets provided by the Italian Ministry for Agricultural, Food and Forestry Policies, which kindly gave the authors the permission to incorporate slightly modified (for purpose of confidentiality) subsets of these datasets into the VMSbase package. These datasets can be used to explore and test the package. In the following sections, these example datasets are also used to describe software functionalities through a typical processing flow. Two small sample datasets are provided: one for VMS data and one for Logbook. Both datasets contain data referring to the activity of 13 vessels in June 2012. In addition, and simply to inform the reader about the computation performance of the package, the time required to complete the steps of a classical flow processing on two large database containing 10^5^ and 10^6^ VMS pings for 1600 fishing vessels, respectively, was measured and reported on one Logbook database containing 194998 records. For the sake of comparison, it is specified that these analyses were performed using a PC with the following characteristics: Intel Core i5 CPU 530 @ 3.20 GHz × 4, 64-bit architecture, 3.8 Gib RAM, Linux – Ubuntu 13.01.

### VMS Data Managing, Cleaning and Enhancing: from Raw Matrices to Database

In the VMSbase, the first step after data loading consists of the creation of a database. This step is performed according to SQLdf R package procedures [11]. All the VMSbase functions are designed to handle this type of database because it strongly increases both the efficiency and speed of loading or storing of data, and in particular when large datasets are used (as commonly happens for VMS/Logbook analyses performed on a large scale or over long periods). The conversion of raw data loaded into Structured Query Language (SQL) databases can be done simply by using the button “Create DataBase” in the “VMS Data Management box” on the main interface.

It is well known that native (that is, stored in and transmitted by the competent national organisms) VMS data are characterized by a certain and variable percentage of erroneous entries [5]. The main types of error can be listed as pings belonging to the following categories: 1) unreasonable positions (e.g. on land); 2) unreasonable speeds (e.g. negative or too high values); 3) unreasonable heading (i.e. values outside a compass range). A final but distinct category simply comprises duplicate records. Intuitively, the pings belonging to one or more of these categories should be removed or flagged. It is found that the permanent removal of records is rarely a good choice. As a consequence, it was decided to flag irregular pings in the dataset. This is possible by clicking on the “Clean DB data” button on the “VMS Data Management box” in the main interface (Figure 1). All the flagged pings are marked as a source of irregularity; the user can decide whether to retain the flagged pings or to remove (part of) them.

The subsequent steps involve the partitioning of VMS ping series belonging to the same vessel in tracks, that is series of pings belonging to the same trip starting from and ending in given harbours.

Given a static map of the harbours in the form of a shapefile or an R dataframe, VMSbase draws a circular buffer around each harbour: the VMS pings falling within the buffers are classified as “in harbour pings”. They will then be associated with the respective harbours. By default, the radius of the buffer is 2 km, although it is customizable by the user. Typically, raw tracks are quite unsatisfactory for performing any kind of analysis, as the frequency of the pings is typically irregular, and in the vast majority of the cases no ping is available in the departure and arrival harbours. For this reason, the package enables the harbours to be inferred and interpolated to a temporal frequency selected by the user. These steps can be performed by clicking on the respective icons in the “VMS Data Management box” in the main interface (Figure 1).

**Figure 3:**
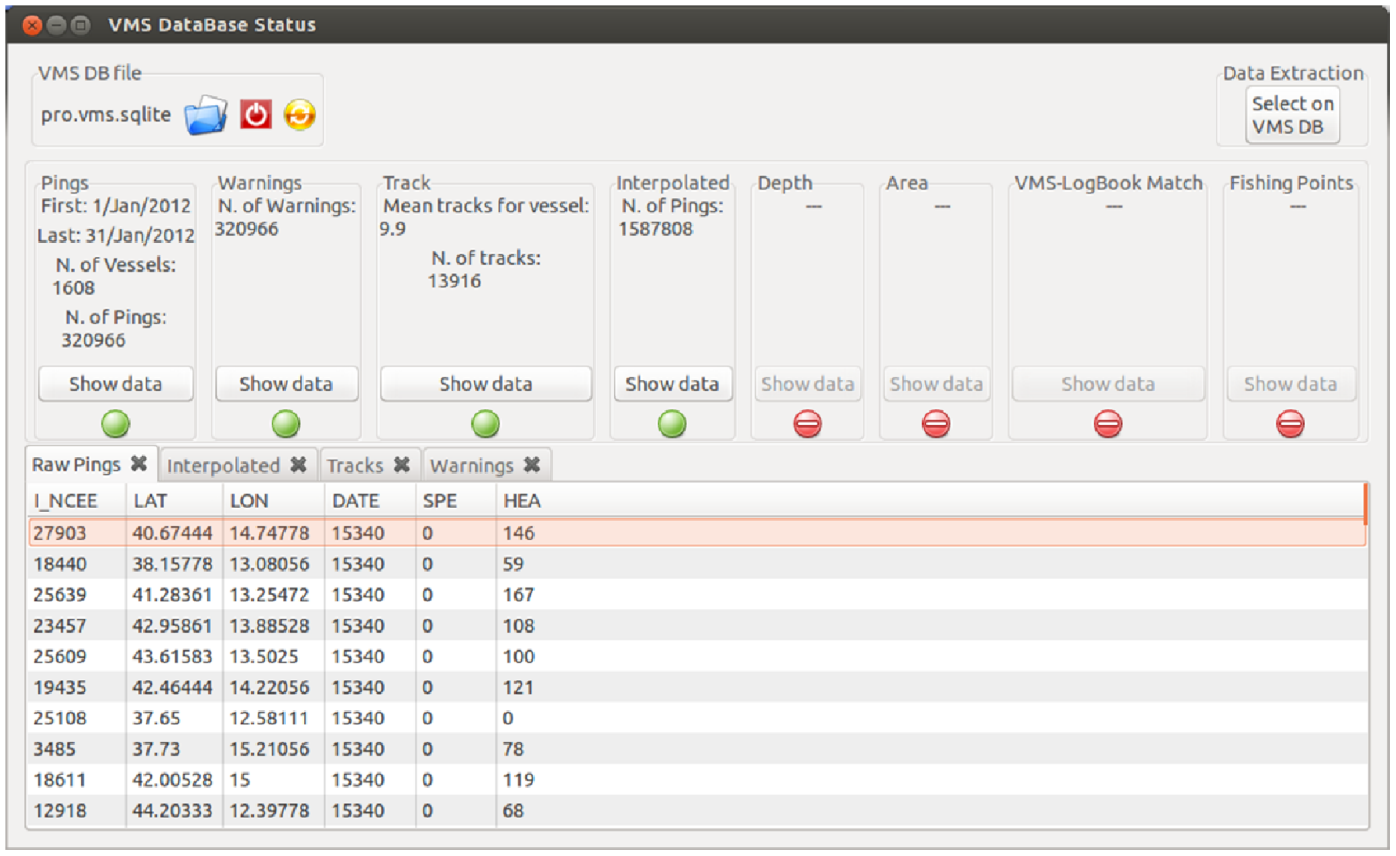
VMSbase interfaces for VMS data viewer or database query. The upper area returns a series of statistical data for the selected database. A traffic light system is present to indicate the analyses already completed (green lights) and the ones which can still be performed (red lights). The bottom area shows the appearance of the dataset. Different panels are used for the native VMS pings, the Tracks (i.e. groups of VMS pings), the Interpolated tracks, the Warnings, etc. The names of the datasets as well as the data in the figure are just for the sake of illustration.

As these steps progressively modify the final database, it is important to graphically explore and follow the analyses through an interface: this is the purpose of the VMS Data Viewer which can be activated by the corresponding icon on the “VMS Data Management box” in the main interface (Figure 1). This tool is designed to show the actual appearance of the database being analysed. The boxes in the upper area of the window (Figure 3) display the status of the analysis by a series of traffic light symbols highlighting which analyses have been already performed, as well as some simple statistics about pings and tracks. A preview of the database is presented at the bottom of this window, organized into different sheets according to the specific analysis step. For instance, it is possible to inspect the warning codes flagging the different error conditions described above, or distinguish between in-harbour and at-sea pings, or lastly between real and interpolated pings. In addition, this interface allows queries to be submitted to the database in order to select subsets of data. For instance, users can select data for a given group of fishing vessels, or within a specific temporal or spatial window.

Examining the algorithms used to implement these steps, it must be said that the assignment of each ping to the “in-harbour” status requires geo-referenced accessory information about harbour positions. While these can be provided in a standard ESRI shapefile, the VMSbase website provides supporting datasets for Mediterranean and World harbours.

If the user does not specify otherwise, the analysis proceeds by ruling out erroneous pings and retaining just two in-harbour pings (one at the beginning and one at the end, respectively, of each track). The partitioning procedure that cuts the database into tracks is based on both detection of in-harbour positions and on a simple filter of delay among successive pings: if the temporal distance among two successive is larger than a threshold value (customizable by the user) the software cuts the series.

The interpolation step warrants a separate description. The term interpolation refers to the artificial increase in VMS ping temporal frequency obtained by applying a mathematical algorithm that estimates position, speed and course at times that are not recorded. In general, this step is needed in order to ensure fisheries data from VMS have proper temporal detail, as the standard frequency of native data is generally low (i.e. around 2 hours). VMSbase is equipped with an algorithm that is, to our knowledge, the one characterized by the lowest level of error and the widest range of applicability in term of fishing activities [8]. This procedure has two features that merit attention: 1) the frequency at which interpolation must be performed can be selected by the user; 2) when applied to a given dataset, the procedure returns pings/tracks aligned to a specific time scale, so that the user can get a “snapshot” of all the vessel’s positions at any given instant of time. In practice, the interpolated database contains pings that are temporally aligned with respect to the time scale, as if all the vessels had sent their signals simultaneously. The resulting dataset can be very useful in estimating e.g. behavioural models, for example, where the interaction between ships is important. Another significant point is that the interpolation step does not require any arbitrary parameter tuning.

**Figure 4:**
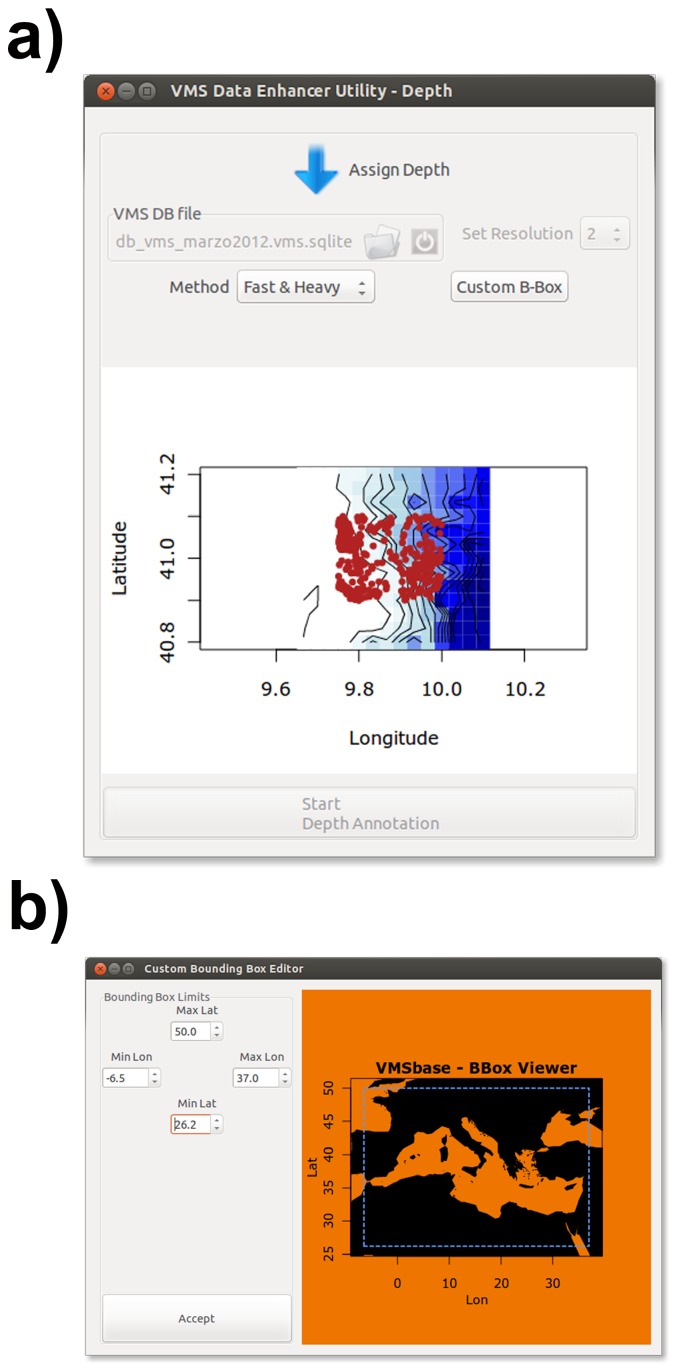
VMSbase interfaces for the Assign bathymetry tool: a) main interface that allows data and algorithm to be selected; b) interface for Custom Box selection of data.

The VMSbase package offers the possibility to enhance VMS data by estimating the sea bottom depth for each ping and computing the bottom sea topology and the isobars for the inspected area. This can be done by means of the algorithms linked to the icon “Assign bathymetry” and “Get Isobaths” in the “VMS Data Management box” in the main interface (Figure 1). The Assign bathymetry tools visualizes the in-progress analysis by a dedicated viewer (Figure 4a) tool and allows applying one of two alternative algorithms: the first one, called “Slow & Light”, is not very fast but can be used by not very powerful computers, whereas the “Fast & Heavy” algorithm allows completing this step of the analysis in about the half of the time but requires computers with at least 8 Gb of Ram. Among others, the package uses the functionalities of the package marmap [12] and allows refining the area of interest through a visual selection box (Figure 4b).

Finally, VMSbase allows each track to be assigned to one of several areas of interest using the standard point-in-polygon function provided in the PBSmapping package [13]. More in detail, the focal point of each track is computed as the mean coordinates of the VMS pings in the track, and the area of interest is established as the one in which the focal point falls.

This procedure requires geo-referenced accessory information about the spatial partitioning of interest. While these can be provided in standard ESRI shapefile, the VMSbase website provides supporting datasets for Mediterranean harbours, Mediterranean Geographic Sub Areas (GSA), ICES rectangles and Large Marine Ecosystems (LME).

### VMS Data Inspection: the Viewers

**Figure 5:**
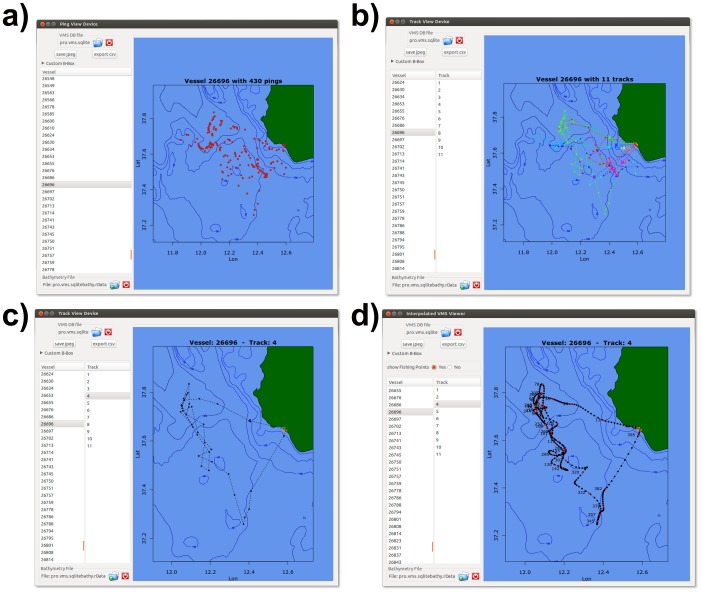
VMSbase interfaces for a) the ping viewer. Single VMS positions are represented by red points; b) the track viewer in which all the tracks for a single vessel are plotted; c) the track viewer in which only a single track for a single vessel is plotted; d) the interpolation viewer in which a single track is plotted, with black points representing interpolated pings and red points representing real pings. It should be noted that, in all cases, the main isobaths are visualized as computed by the “Get Isobaths” tool in the main interface. The names of the datasets as well as the data in the figure are just for the sake of illustration.

Users familiar with processing VMS data know that visual inspection and control of data flow is desirable and sometimes necessary. The VMSbase package is equipped with four different viewer tools allowing data to be visually inspected at different stages of the analysis. The simplest of these tools is the Ping Viewer, accessed through the button of the same name on the VMS Data Visualization box on the main interface (Figure 1). This viewer (Figure 5a) is designed to show the pings within a cleaned database, before partitioning the pings into tracks, and then simply allows subsets of pings to be selected with reference to the Vessel identity (UE Number). The aim of this tool is to provide the graphical support for a first inspection of uploaded data.

Conversely, the Track Viewer (accessible through the corresponding icon on the main interface – Figure 1) allows single tracks belonging to each vessel in the cleaned database to be visualized (Figures 5b and 5c). This can be useful in inspecting vessel activity with respect to the harbours, while it also allows the whole path travelled by a given vessel on a given fishing trip to be observed.

The Interpolation Viewer is conceived to show the interpolated tracks in which different colours represent native (real) and interpolated pings. This would allow the user to inspect the soundness of the interpolation step (Figure 5d).

A particular mention is needed for the “Google viewer” tool. It allows the VMS data to be visualized on Google Viewer [14], so that the user can produce easy to interpret and more realistic visualization of both fishing effort and effort behaviour. A representative sample of the maps produced by this tool was not included in the present paper due to copyright restrictions of the journal, which is not permitted to publish most satellite images or third-party maps. However, samples can be found on the VMSbase website (www.vmsbase.org).

Each of the VMSbase viewers (with the only exception of the Google Viewer) allows saving the image as a jpeg file and exporting plot data as a csv file.

### Logbook Data Managing and Cleaning: from Raw Records to Database

VMSbase allows Logbook data to be handled in a way that is similar to (and consistent with) the procedure described above for VMS data. Logbook data can be uploaded using an interface (accessed using the icon Edit Raw Data in the Logbook Data Management frame located in the right hand side of the main interface – Fig. 2b) which asks the user to select the mandatory fields (columns) in the raw file to be uploaded and the field separators (comma, semicolon and tabulation). Mandatory columns for Logbook are: Vessel UE Code, Departure Data & Time, Arrival Data & Time, Species, and Catch quantity. In addition, users can directly upload information about used gear and/or métier (see next section) as optional fields. However, the interface allows data to be uploaded in the standard EFLALO formats for compatibility purposes. VMSbase uses the 3-alpha standard FAO code for species name (ftp://ftp.fao.org/FI/STAT/DATA/ASFIS_sp.zip) throughout the analyses, and so the input data must conform to this standard in order to be correctly uploaded.

**Figure 6:**
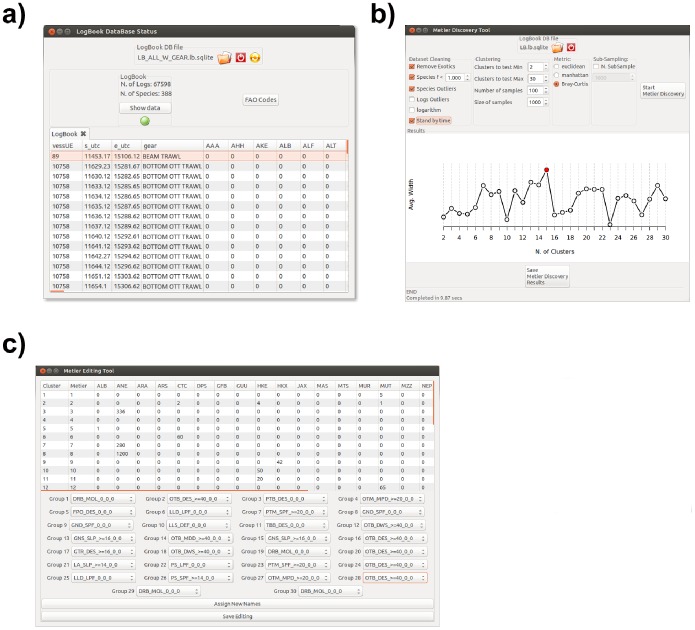
VMSbase interfaces for a) Logbook database viewer, the tool to visually inspect the logbook data; b) Métier discovery, the tool for searching the métiers, defined as catches profiles, in a given logbook database; c) Métier editing tool, which allows the user to assign DCF labels to the detected métier. The names of the datasets as well as the data in the figure are just for illustrative purposes.

Uploaded data is then converted in a SQL database via the Create Database icon, and the rest of the analyses are performed on database objects. Raw logbook data to be uploaded are generally organized as a matrix with fishing trips in a row and a single column for each species, so that there are multiple rows for a single fishing trip, one row for each captured species. During database creation, the uploaded logbook data are converted into a matrix with a single row for each fishing trips and the same number of columns as the total number of species in the uploaded dataset, plus the columns for gear and métier (if any). This structure of logbook data is consistent with the subsequent analyses. The generated database can be visualized and inspected using the Logbook Data Viewer, which is accessible through the related icon in the main interface. This viewer provides some basic statistics (number of records, number of species), and, after métiers assignment, reports the category to which each logbook record belongs (Figure 6a).

### Métier Identification and Classification

A major topic of fishery management and a mandatory rule within the Data Collection Framework for the European Union (DCF, [15]) is the identification of groups of fishing trips, usually referred to as a “métier” [16], characterized by the same exploitation pattern (e.g. gear used, fishing ground, target species). This feature is found especially in multi-species, multi-fleet fisheries such as those in Mediterranean Sea, in which an array of species is exploited by different gears or fishing techniques [9]. The DCF established a hierarchical scheme for the identification of métiers according to gear used and target assemblage (https://datacollection.jrc.ec.europa.eu/web/dcf/wordef/fishing- activity-métier). If the raw logbook data contains information about gear and/or métier, the user can skip this step of the analysis. Otherwise logbooks, as simple catches profiles without information about used gear, do not directly allow the DCF métiers of level 5–6 in which a set of fishing trips falls to be established, and different approaches have been developed to assign logbook observations, that is catches profiles, to one of a list of *a-priori* defined métiers. As a general rationale, these approaches use a clustering procedure that could either be preceded by a multivariate analysis aimed to reduce the number of descriptors, that is the species captured during each fishing trip and recorded in the logbook [17]–[19], and then reduce the computational burden related to the clustering step, or directly applied to whole (unreduced) dataset [20]. Among others, the Clustering Large Applications (CLARA) procedure, a partitioning technique specifically designed to manage very large data sets, has proved to be useful and sound in this context [21]. The CLARA procedure implemented in the R package “cluster” [21] is used in VMSbase to identify métiers throughout the logbook database, without any preliminary reduction of data dimensionality (Figure 6b). However, the native CLARA function, which is hardcoded in C language, was further enriched by the introduction of the Bray-Curtis distance. This function was also made visible to the user, as we think that it can be useful in many analyses, even outside the study of VMS data. The method also allows the assessment of the optimal number of groups (métiers) in the dataset by the analysis of the “average silhouette width” (ASW - [22]).

The “Métier discovery” tool in VMSbase allows the number of métiers in a given logbook database to be detected and their characteristics in terms of catches for the centroid of each métier/cluster. Different options are available in this interface to handle input data (e.g. to remove outliers or standardize per trip temporal length) and perform the clustering, including parameters for the CLARA algorithm (i.e. number of random samples and size of each sample) and distance to be used (between Euclidean, Manhattan and Bray-Curtis). At the same time, the interface contains a graphical area in which the silhouette profile for different clustering is progressively updated during the search phase. At the end of the search phase, a list of candidate métiers with related catches profiles is listed and can be saved. The user is required to inspect this list and recognize the different DCF métier, which will be definitively associated with the catches profiles. This is possible via the “Métier Editing Tool” (Figure 6c). This step can be skipped by using the reference métier profiles natively distributed with the VMSbase package. These consist of a set of 18 profiles, corresponding to the main typologies of fishing activity exerted by the Italian fishing fleet in the Mediterranean, and were obtained in an independent study which is in preparation. However, after identification and labelling of the métier, the classification obtained can be used to classify all the records in the logbook database. This essentially consists in assigning each record in the logbook database, and eventually new records subsequently added, to one of the identified métiers. This step is performed in VMSbase by means of a fuzzy membership function [23], [24] that represents a modification of the common crisp approach for CLARA, in which the rule is allocation to the nearest medoid.

### Matching between VMS and Logbook Data and Identification of Fishing Set Positions

Having processed both VMS and Logbook data separately, the user normally has complete information about a vessel’s course at sea, that is information about positions, speed and heading (from VMS) and about the gear used and resources targeted. The merging of this information opens up the possibility of spatially and temporally resolving the various fishing activities characterizing a given fleet [2], [3], [25]. The critical step in this framework is the assignment of each VMS track (that is, each fishing trip) to the respective record in the logbook (if any). This is primarily possible because records in both VMS and Logbook dataset bear the Vessel UE Code. Secondarily, the temporal limits of each VMS and Logbook record must be considered. VMSbase identifies the pairs of VMS and logbook records by: 1) computing, for each record in each dataset, the temporal interval of fishing activity by its extreme UTC values using the format defined in the R package “intervals” [26]; 2) using these intervals to find overlapping records between VMS and Logbook database; 3) in the case of multiple overlaps (e.g. more VMS tracks overlapping the same logbook record) each VMS track is assigned to that métier; 4) in the case of ambiguity (e.g. multiple logbook records for a given track) the VMS track is coupled with the logbook record characterized by the largest overlap. This procedure is implemented in the “LogBook-VMS Matching Tool”. At the end of this procedure, the VMS database is updated with the information referring to métier, and a new sheet is made available in the VMS Data Viewer. This sheet reports the list of matched tracks.

**Figure 7:**
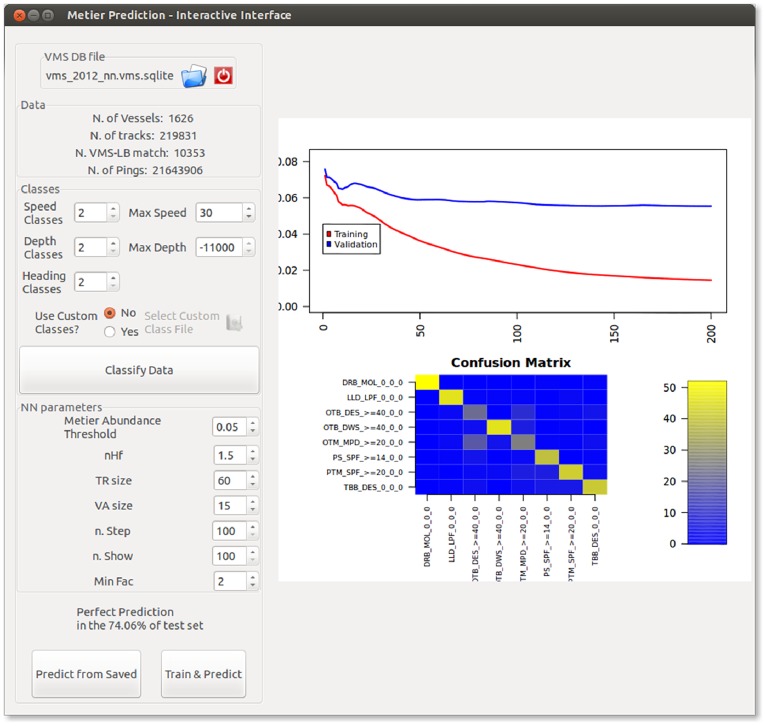
VMSbase interface for Métier Prediction tool. It allows customizing the parameters of the Artificial Neural Network and then train it, but also shows the performance of the trained ANN in terms of correct prediction and allows loading a previously trained ANN and applying it on a new dataset.

However, it often happens that logbook records are not available or may not contain enough information to infer the métier for certain VMS tracks [16], [27]. In this case, the use of VMS data for detailed analyses of fishing effort could be hampered. VMSbase offers a tool to overcome this drawback: it supplements the Artificial Neural Network approach described in [9]. This approach is accessible via the “Predict Métier” tool in the main interface (Figure 7). This tool exploits a database in which the métiers have been assessed by means of logbooks for some tracks as training dataset, and then it returns the métiers for all the remaining tracks by predicting them from VMS data. A detailed description of the rationale and of the methodology is reported in [9].

**Figure 8:**
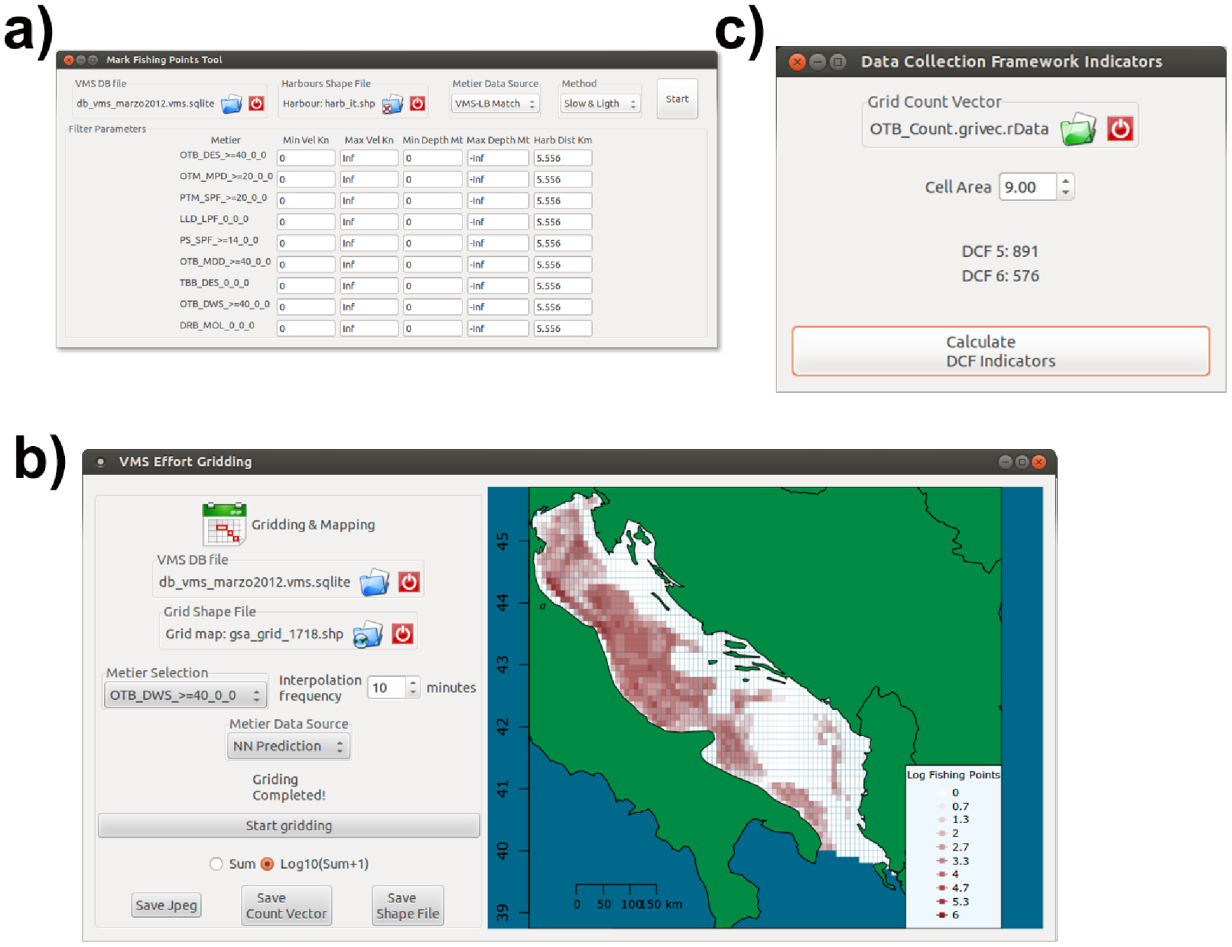
VMSbase interfaces for a) “Mark fishing points” tool, which allows the user to specify the speed and eventually the bathymetric range for different fishing activities; b) “Effort Gridding”, the tool designed to associate fishing effort with grids or other partitioning, which are then plotted and exported in the desired formats. This tool returns a value (hours of fishing effort activity) for each cell of the grid, while the results can be saved and exported as ESRI shapefile or CSV textfile; c) “DCF Indicators”, the tool aimed to compute the values for DCF indicators of fishing pressure 5 and 6.

At this point, it is possible to distinguish between the different phases that normally comprise a fishing trip: drifting, fishing and steaming [28], [29]. However, this analysis is often reduced to the distinction between fishing and non-fishing set positions, which is generally performed using a criterion based on vessel speed and then assigning native or interpolated VMS pings to one of these two classes by comparing their values of speed to a previously defined set of thresholds [5]. This stems from the consideration that the fishing vessel’s speed profile is characterized by a multi-modal distribution of values, with three modes corresponding to drifting (speed values around zero km/h), fishing (positive speeds, with intermediate values) and steaming (speed values near the maximum allowed by the engine and other technical characteristics of the vessel). VMS pings with speed values between the minimum and maximum threshold for the “fishing” mode are therefore assumed to be fishing set positions. VMSbase follows this rationale, but allows using a combination of different thresholds: one for speed, one for depth and one for the distance from harbour (Fig. 8a). It should be noted that the possibility of setting a range for depth depends upon the fact that VMSbase allows the sea bottom depth corresponding to each ping in the VMS database to be assessed. The introduction of these criteria, which however represents an option for the user, is justified by the consideration that different types of towed gear are typically used in relatively well defined bathymetric range, and so this option could lead to a better estimate of the fishing pressure.

### Mapping Fishing Pressure and Computing Pressure Indicators

If all the previously described analyses are applied to a set of input VMS and logbook data, it is possible to obtain a fully processed database, which contains information about vessel course (by interpolated VMS pings), activity (as métier by logbook data), area of activity (e.g. GSA), and sea depth. All this information is disaggregated at the track level so that each record in the final database is a single fishing trip. These data can be then used to plot the fishing effort, which is typically computed as the amount of fishing effort deployed in each element (e.g. cell) of a given space partitioning (e.g. grids). To perform this analysis on VMSbase, it is possible to use the “Gridding & Mapping” Tool (Figure 8b), which analyses a given database and provides the count of fishing set positions for each element of the selected space partitioning, which can be uploaded as an ESRI shape file (.shp). The output of this tool is both graphical, as a map is produced to visualize the results, and numerical: following the user’s instructions, the tool can either update the input shapefile by adding a new field with the per element counts, or save an R vector with the count values.

VMSbase also allows computing two of the DCF indicators aimed at assessing the level of fishing pressure for a given area of interest. These two indicators, respectively named “Indicator 5 - Distribution of fishing activities”, and “Indicator 6 - Aggregation of fishing activities” are designed to quantify the fishing pressure as the amount of area disturbed by a specific fishing activity in a defined temporal scale (generally a month). The values of these indicators are computable by the “DCF indicators” tool (Figure 8c). More in detail, the indicator 5 is computed after association of fishing set position with the cell (by the “Gridding & Mapping” tool described above) as the total area of cells in which fishing effort is allocated, while the indicator 6 is computed as the total area of cell scoring 90% of total observed fishing effort [4].

Finally, the VMSbase package provides a tool for analysis and visualization of “Trawled Area”. This tool is devised to handle data about fishing gear characteristics for each vessel (e.g. size of gear mouth for trawl and other towed gears, number of gears deployed for beam trawl, etc.). These data are used together with VMS and logbook data to produce an evaluation of the fishing effort as the effective trawled surface, which is computed for each fishing track as the length of the vessel trajectory multiplied by the overall width of the gears (OWG). While OWG can be basically computed as the width of a single gear (es. a bottom trawl net) multiplied by the number of gears simultaneously deployed (such as in the case of beam trawl), the user can specify whatever function he chooses OWG more accurately, for instance a function relating OWG to the power or length over all (LOA) of the fishing gear, or to the sea depth at which fishing activity is carried out. This tool is implemented basically but requires external data (e.g. fleet register of more refined information). For this reason, and also due to the fact that a reliable relationship for computing OWG is not available for the Italian fleet, this tool was not included in the analyses presented in this paper.

## Results

In this section we briefly report the results of the performance tests carried out on the two sample datasets containing 10^5^ and 10^6^ VMS pings for 1600 fishing vessels, respectively. Although these datasets comprise the activity of the Italian fleet for the first few months of the year 2012, no conclusions or indications are drawn on the basis of actual patterns observed.

The performances (time required for computation) of VMSbase package in completing the different steps of a typical processing flow and the main related statistics are reported in Table 1.

**Table 1:** Temporal performances (time required for computation) for each step of a typical data flow.

		Dataset #1∶10^5^ initial VMS pings		Dataset #2∶10^6^ initial VMS pings	
General Step of the Analysis	Tool	Time required tocomplete the processing(minutes)	Main Statistics	Time required tocomplete theprocessing (minutes)	Main Statistics
**VMS Data** **Management**	Edit RawData	<1	0 NAs found in latitude degrees;0 NAs found in latitude minutes;0 NAs found in latitude seconds;0 NAs found in latitude direction;0 Latitudes out of range (−90/90);0 NAs found in longitude degrees;0 NAs found in longitude minutes;0 NAs found in longitude seconds;0 NAs found in longitude direction;0 Longitudes out of range (−180/180);0 NAs found in dates; 0 NAs found inhours; 0 NAs found in minutes; 0 NAsfound in seconds; 0 dates found withbad format; 0 NAs found in knotsspeed; 0 NAs found in degrees heading	<1	0 NAs found in latitude degrees; 0 NAs found inlatitude minutes; 0 NAs found in latitude seconds;0 NAs found in latitude direction; 0 Latitudes outof range (−90/90); 0 NAs found in longitude degrees;0 NAs found in longitude minutes; 0 NAs found inlongitude seconds; 0 NAs found in longitude direction;0 Longitudes out of range (−180/180); 0 NAs found indates; 0 NAs found in hours; 0 NAs found in minutes;0 NAs found in seconds; 0 dates found with badformat; 0 NAs found in knots speed; 0 NAs found in degrees heading
	CreateDatabase	<1	/	<1	/
	Load DBin theVMS DataViewer	<1	/	<1	/
	Clean DBData	20	Found 4584 (4.58% of total) duplicatedpings; Found 31019 (31.02% of total)pings in harbour; Found 7550 (7.55%of total) pings on land; Found 38(0.04% of total) not coherent pings	150	Found 98381 (9.84% of total) duplicated pings;Found 272996 (27.31% of total) pings in harbour;Found 124015 (12.41% of total) pings on land;Found 279 (0.03% of total) not coherent pings
	Track Cutting	5	3883 tracks detected	40	36994 tracks detected
	Interpolation(10 minutesfrequency)	20	Number of pings changed increasesfrom 10^5^ (real) to 4.9*10^5^ (interpolated)	180	Number of pings changed increases from 10^6^(real) to 4.4*10^6^ (interpolated)
	Assign Bathymetry(1 degree resolution)	150 (Fast& Heavy Algorithm)/60(Slow & Light)	/	1000 (Fast& HeavyAlgorithm)/400(Slow & Light)$	/
	Assign Area(MediterraneanGSAs)	100	/	900	/
	**Subtotal**	**295 (∼5 hours)**		**2237**	**2270 (∼37 hours)**
**Logbook Data** **Management** **(2×10^5^ initial** **records)**	**Tool**	**Time required to complete the** **processing (minutes)**	**Main Statistics**		
	Edit Raw Data	<1	20 NAs found in Start Times; 140 NAs found in End Times;0 NAs found in Species; 0 NAs found in Quantity;Removed 0.08% of data, that is 160 logbooks		
	Create Database	180	This step requires only few minutes for the EFLALO format		
	Métier Discovery(searching between2–30 groups on thewhole dataset with100 samples of 1000records each)	20	The best partitioning corresponded to 11 métiers. These are alsoavailable into to the package as reference dataset for Métier Classification		
	Métier editing	Depends by the user, reasonablyno more than half an hour	69397 records in the database; 387 species		
	Métier Classification	20	/		
	**Subtotal**	**∼250 (∼ 4 hours)**			
**VMS-Logbook** **Analysis**	**Tool**	**Time required to complete** **the processing (minutes)**	**Main Statistics**	**Time required to** **complete the** **processing (minutes)**	**Main Statistics**
	Logbook- VMSMatching	5	60.3% of VMS tracks with matching in LB database	30	57.2% of VMS tracks with matching in LB database
	Predict Métier: DataPreparation	10	/	50	/
	Predict Métier:Training ANN	3	Prediction completed for all the tracks without métiers by LB database	3	Prediction completed for all the trackswithout métiers by LB database
	Find Fishing Points	30	/	45	/
	**Subtotal**	**∼50 (∼1 hours)**	**∼128 (∼2 hours)**		
**Data Output**	**Tool**	**Time required to complete** **the processing (minutes)**	**Main Statistics**	**Time required to** **complete the processing** **(minutes)**	**Main Statistics**
	Gridding & Mapping(for each métier)	2	/	9	/
	DCF Indicators(for each métier)	<1	/	<1	/
	Trawled Area Viewer	Untested	/	/	/
	**Subtotal**	**∼3**	**∼10**		
**Complete data** **flow**	**Total**	**∼600 minutes (∼10 hours)**	**∼3180 minutes (∼53 hours/2.5 days)**		

These data was measured on three sample datasets (two for VMS and one for Logbook).

$ The Fast & Heavy Algorithm was tested on a different personal computer with 16 Gb of Ram.

The initial raw dataset for VMS data was uploaded, transformed into an SQL database and then cleaned. The cleaning step flagged a total of 12.17% and 22.28% of the initial pings. Then, 3883 and 36994 tracks (fishing trips) were identified. These tracks were interpolated at a frequency of 10 minutes and matched with the Logbook database, which contained 69397 records (Logbook events) for 387 species. The Logbook database was larger than the two VMS databases since we used the Logbook dataset for the whole 2012 activity of the Italian fleet, in order to better test the package performance. 60.3% of the tracks for the 10^5^ VMS dataset and 57.2% of tracks for the 10^6^ VMS dataset matched Logbooks records. The “Métier Discovery” tool allowed a list of 10 métiers to be identified which were assigned to the DCF métier in Table 2 by observing the catches profiles. The complete procedure for Logbook data uploading, cleaning, Métier Discovery and classification required around 4 hours, but this value is reduced considerably if an EFLALO dataset is used as input. The original Italian format actually requires a complex transformation phase in order to be used.

**Table 2:** List of métiers identified in the sample Logbook database for the activity of the Italian fishing fleet during year 2012.

DCF Code	Extended definition
DRB_MOL_0_0_0	Boat dredge for Molluscs
LLD_LPF_0_0_0 SWO	Drifting longlines for large pelagic fish
OTB_DES_> = 40_0_0	Bottom otter trawl for Demersal species
OTB_DWS_> = 40_0_0	Bottom otter trawl for Deep Water species
OTB_MDD_> = 40_0_0	Bottom otter trawl for Mixed demersal species and deep water species
OTM_MPD_> = 20_0_0	Midwater otter trawl for Mixed demersal and pelagic species
PS_SPF_> = 14_0_0	Purse seine for small pelagic fish
PS_LPF_> = 14_0_0	Purse seine for Large pelagic fish
PTM_SPF_> = 20_0_0	Pelagic pair trawl for Small pelagic fish
TBB_DEF_0_0_0	Beam trawl for Demersal species

The tracks matched to logbooks were directly assigned to the corresponding métiers, the tracks without such matching were processed via the Artificial Neural Network in the “Predict Métier” tool. After this, all the tracks in the VMS database were assigned to one of the métiers.

The entire processing flow for VMS data matching with Logbook and eventual prediction of métier required between 1 and 2 hours for the two datasets.

The whole data processing, from raw VMS and logbook input data to the outputs (mapping or DCF indicators computation) required 10 and 53 hours for the two VMS datasets, respectively.

It seems that the critical steps, in terms of time required for computation, are (in order of size): 1) the assignment of the estimated values of bathymetry to each VMS database pings (around one minute for a dataset of ∼3300 pings); 2) the Assignment of Area to each VMS database track (around one minute for a dataset of ∼5000 pings); 3) the Creation of Logbook database (around one minute for a dataset of ∼400 records); 4) The interpolation (around one minute for a block of ∼25000 pings). However, the Fast & Heavy algorithms available for the Assignment of Bathymetry can significantly reduce the time required for computation. Each of the other steps does not require more than 30 minutes for both VMS datasets. It is important to stress that the interpolation procedure significantly increased the number of pings, which passed from 10^5^ to 4.9*10^5^ and from 10^6^ to 4.4*10^6^.

## Discussion

The usefulness of VMS data as proxy for fishing effort has been repeatedly demonstrated in recent years: they have been used to assess physical effects of fishing gears on sea bottom [30], to relate fishing effort pattern to catches and landings composition [2], [31], for marine conservation purposes [32], and to build predictive models for fisheries management [4], [33], [34]. However, while an adequate suite of tools is required in order to maximize this source of information, the development of methodologies cannot neglect the peculiarities of the different fishing systems characterizing each country and/or environmental context. The fisheries in the Mediterranean Sea and Black Sea, for instance, have a distinctive multi-gear and multi-species nature which represents an hindrance in analysing the real pattern and impact of fishing [7] and requires appropriate methodologies [33]. Furthermore, it could be argued that there is still no methodological platform to support long-term collection, processing and management of VMS data, even in combination with logbook data. Indeed, VMStools [5], which represents a first step in this framework, is primarily designed to handle ICES data formats (i.e. EFLALO and TACSAT) and to perform analyses for North Sea fisheries. This study introduces VMSbase, which is a software platform specifically devised to take into account the peculiarities of virtually any kind of fisheries in terms of both data formats and technical/dynamic aspects of fisheries such as vessel behaviour. An example in this sense is the “Predict Métier” function, based on Artificial Neural Network and is devised to learn how to associate vessel behaviours (as captured by VMS) with the DCF classification of fishing activities by gear and target assemblages (the métiers).

In the meantime, users can build and feed a database system, which guarantees coherent storage and management of the data, also facilitating the process of querying for *ad-hoc* studies. In fact, the data architecture of data in VMSbase is based on SQL, one of the most powerful and common database structures, which has been proposed and applied in fisheries contexts [2], [35], [36]. This also implies that SQL databases produced by VMSbase can be handled by several software products, most of which are freeware, hence ensuring interoperability between different operating systems (i.e. Windows, Linux, Mac OS).

The core idea of VMSbase is to use R potentialities through a widgets interface and to handle VMS/Logbook data as a database. Moreover, VMSbase offers the opportunity to expand the information delivered by VMS data by integrating environmental aspects (i.e. by estimating the sea bottom depth for each VMS ping) and using these data for more refined assessments (i.e. the speed for distinguishing between steaming and fishing pings). This represents a desirable but hitherto unexplored development, since VMS pings are, in essence, positions on the sea surface that can be easily linked to any kind of spatial pattern whatever (e.g. sea surface temperature or nutrients concentrations).

VMSbase works as a classical R add-on package that comprises a series of functions and routines designed to enhance input data. This implies that VMSbase can be executed by the R-console command line. But VMSbase also offers the opportunity to work with a user-friendly graphical interface that: 1) allows users without an extensive knowledge of R to easily use the package tools; 2) enhances the analyses by allowing visual inspection of data via the broad selection of plotting interfaces; 3) allows users to manage their workflows as a project, by defining from the outset of the procedure the desired accessory information (i.e. bathymetry, space partitioning such as GSA or others, etc.). Obviously, VMSbase code is completely open and distributed under a GPL2-type license (http://www.gnu.org/licenses/gpl-2.0.html).

While some of the VMSbase tools are at least partly original (namely the Interpolation and the Métier classification), the package relies on several other R packages (Table 3). The first one of these original aspects (the interpolation algorithm) is extensively described in [9]. The Métier classification is original in the sense that: 1) it uses the well-known CLARA algorithm but integrates the Bray-Curtis distance [37]; 2) a preliminary multidimensional analysis (i.e. PCA) is not required.

**Table 3:** List of dependencies (other R add-on packages) for VMSbase.

Package Name	Scope	Reference
CairoDevice: Cairo-basedcross-platform antialiasedgraphics device driver	Cairo/GTK graphics device driver withoutput to screen, file (png, svg, pdf, and ps)or memory (arbitrary GdkDrawable orCairo context).	[46]
chron: Chronological objects whichcan handle dates and times	Chronological objects which can handledates and times	[47]
cluster: Cluster Analysis ExtendedRousseeuw et al	Cluster Analysis, extended original fromPeter Rousseeuw, Anja Struyf and MiaHubert.	[21], [48]
Ecodist: Dissimilarity-based functionsfor ecological analysis	Dissimilarity-based analysis functionsincluding ordination and Mantel testfunctions, intended for use with spatialand community data.	[49]
Fields: Tools for spatial data	Fields is for curve, surface and functionfitting with an emphasis on splines, spatialdata and spatial statistics. Implementationof sparse matrix methods for large data setsand currently requires the sparse matrix(spam) package for testing and use withlarge data sets.	[50]
Ggmap: A package for spatialvisualization with Google Mapsand OpenStreetMap	Visualization of spatial data and modelson top of Google Maps, OpenStreetMaps,Stamen Maps, or CloudMade Maps usingggplot2.	[14]
Gwidgets:gWidgets API for buildingtoolkit-independent, interactive GUIs	Toolkit-independent API for buildinginteractive GUIs.	[51]
GWidgetsRGtk: Toolkit implementationof gWidgets for RGtk2	Port of gWidgets API to RGtk2	[52]
Intervals: Weighted Logrank Testsand NPMLE for interval censored data	Functions to fit nonparametric survivalcurves, plot them, and perform logrankor Wilcoxon type tests.	[53]
Mapdata: Extra Map Databases	Supplement to maps package, providingthe larger and/or higher-resolution databases.	[54]
Maps:Draw Geographical Maps	Display of maps. Projection code andlarger maps are in separate packages(mapproj and mapdata)	[55]
Maptools: Tools for reading andhandling spatial objects	Set of tools for manipulating and readinggeographic data, in particular ESRIshapefiles; C code used from shapelib.It includes binary access to GSHHSshoreline files. The package also providesinterface wrappers for exchanging spatialobjects with packages such asPBSmapping, spatstat, maps, RArcInfo,Stata tmap, WinBUGS, Mondrian, andothers.	[56]
marmap: Import, plot and analyzebathymetric and topographic data	Import, plot and analyze bathymetric andtopographic data	[12]
Outliers: Tests for outliers	A collection of some tests commonlyused for identifying outliers	[57]
PBSmapping: Mapping FisheriesData and Spatial Analysis Tools	Two-dimensional plotting featuressimilar to those commonly availablein a Geographic Information System(GIS). Embedded C code speedsalgorithms from computational geometry,such as finding polygons that containspecified point events or convertingbetween longitude-latitude andUniversal Transverse Mercator (UTM)coordinates.	[13], [58]
Plotrix: Various plotting functions	Lots of plots, various labeling, axis andcolor scaling functions	[59]
Sp: classes and methodsfor spatial data	Classes and methods for spatial data.The classes document where the spatiallocation information resides, for 2Dor 3D data.	[60]
Sqldf: Perform SQL Selects onR Data Frames	Manipulate R data frames using SQL.	[11]

The possibility to use the Bray-Curtis distance is justified by the fact that métiers can differ both in terms of the arrays of exploited species and of relative abundances of these species in the catches. In this way, a measure of distance that quantifies the compositional dissimilarity between two different observations (i.e. two logbook records) based on abundances in each observation, seems to be appropriate. In effect, the Bray-Curtis distance (BCD) between two logbook records (called *i* and *j*) is computed as:
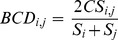
where 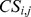 is the sum of the lesser value of catches for only those species in common between both logbook records, while *S_i_* and *S_j_* are the total number of specimens counted at both sites.

This second point deserves more profound examination. Scientific literature contains a flow of studies, starting with the pioneer work of [18], in which the challenge of identifying métiers over large logbook datasets is addressed in a two-step approach: (1) reduction of data dimensionality by a multivariate analysis, typically PCA; and (2) identification of groups of observations by clustering technique. Although the PCA is a technique widely applied to the reduce dimensionality of the logbook dataset, it is worth noting that this scaling approach is affected by a series of issues when applied to sparse matrices, as commonly occurs with logbook, and particularly for fisheries in the Mediterranean Sea. The presence of many zeros can actually indicate the existence of spurious correlations in the results [39]. Furthermore the basic assumptions of a PCA (input data should be multi-normal, or at least approximately symmetric [38]) seem to be unreasonable for a logbook dataset. These issues could be particularly serious if the PCA were computed on the covariance matrix [38]. Consequently, even if a scaling approach allows a relevant reduction of data, and hence a reduction of time required for the analysis, it seems that its adoption does not have a sound theoretical basis. Thanks to the structure of VMSbase package, which is designed to work with an SQL database manager, the use of a filter such as the PCA step becomes unnecessary. Therefore the VMSbase package, by performing the cluster analysis directly on the original data, using the CLARA algorithm, proves to be powerful in analysing large datasets even without a preliminary reduction through a PCA-step [20].

The temporal performances documented using the test datasets indicate that the package is coherent with the general characteristics of the R environment: R is designed to offer a virtually infinite list of free resources developed by its large community, but the R interpreter is not particularly fast [39]. However, the VMSbase performance should be measured against the amount of data processed: in this study we demonstrated that large datasets can be processed in just a few days, and it should be taken into account that these large datasets correspond to the information about the activity of a very large fishing fleet (the Italian fleet accounts for more than a half of the whole Mediterranean fleet).

On the basis of these considerations, we think that VMSbase could be a practical help to scientists and technicians in analysing fishing effort spatial and temporal patterns, which is expected to become one of the main tasks in fisheries science [40]. VMSbase was already used to study the relationship between fishing effort and resource abundance in the Adriatic Sea [41] and in the Strait of Sicily [34]. Further planned developments for VMSbase would concern the integration of other approaches for: 1) catches (logbook) data analysis, and in particular the application of Self Organizing Maps [42], [43]; 2) interoperability with other applications in which the temporal evolution of fishing effort and resources is modelled [34], [44], [45].
